# Effect of Electroacupuncture at Fengchi on Facial Allodynia, Microglial Activation, and Microglia–Neuron Interaction in a Rat Model of Migraine

**DOI:** 10.3390/brainsci12081100

**Published:** 2022-08-18

**Authors:** Pei Pei, Shengwei Cui, Shuaishuai Zhang, Sheng Hu, Linpeng Wang, Wenming Yang

**Affiliations:** 1 Neurology Department, The First Affiliated Hospital of Anhui University of Chinese Medicine, Hefei 230031, China; 2 Anhui University of Chinese Medicine, No. 350, Longzihu Road, Xinzhan District, Hefei 230012, China; 3 Acupuncture and Moxibustion Department, Beijing Hospital of Traditional Chinese Medicine, Capital Medical University, Beijing 100010, China

**Keywords:** electroacupuncture, migraine, microglia, inflammatory cytokines

## Abstract

The purpose of the work was to investigate whether electroacupuncture (EA) could ameliorate migraine central sensitization by modulating microglial activation and the subsequent release of inflammatory cytokines in the trigeminal nucleus caudalis (TNC) in a rat model. Establishment of a rat model of recurrent migraine was achieved through repeated dural electrical stimulation (DES). After nine sessions of acupuncture treatment at Fengchi (GB20), facial mechanical thresholds were measured by electronic von Frey measurements. Microglial activation and cytokine receptors of TNC were evaluated by immunofluorescence staining. The expression of microglial biological marker Ibal-1, proinflammatory cytokines, and cytokine receptors in the TNC were evaluated by Western blot and/or real-time polymerase chain reaction. In addition, the effects of inhibition of microglial activation on facial thresholds and neuronal activation (i.e., expression of c-Fos in the TNC) induced by DES were observed. After consecutive EA-GB20 treatments, the facial withdrawal threshold was significantly higher than in the model group at different time points (*p* < 0.05). The hyperreactivity of microglia induced by DES was significantly inhibited, and the expressions of Ibal-1, interleukin-1β, tumor necrosis factor-α, and their receptors in the TNC were also significantly decreased (*p* < 0.05). Inhibition of microglia by minocycline demonstrated an acupuncture-like role, which was manifested by ameliorated mechanical hyperalgesia and decreased neuronal expression of c-Fos, Iba-1, and inflammatory factors. EA at GB20 could ameliorate migraine facial allodynia by inhibiting microglial activation and the subsequent release of inflammatory cytokines and their receptors in the TNC.

## 1. Introduction

Cutaneous allodynia (CA) is a key characteristic of migraine, a pain perception/response to a nonpainful stimulus, affecting 50~70% of migraineurs [1,2]. It is considered an external symptom of migraine’s central sensitization in trigeminovascular pain pathways and a threat factor for episodic progression to chronic migraine and is related to frequency, disability, severity, and other migraine symptoms [3,4,5]. To date, there have been no specific medications used to inhibit or delay central sensitization, necessitating research into valuable alternative therapies. Acupuncture and electroacupuncture (EA) have been recommended as alternative medicine options to treat migraines; however, there is insufficient data demonstrating the underlying mechanisms, especially the central mechanisms [6].

Previous studies on the mechanisms of chronic migraine have largely focused on the central neuronal sensitization mechanisms, such as the spine/medulla oblongata ascending pathway and brainstem descending regulatory system [7,8]. Recently, studies have begun to focus on the role of microglia in chronic pain and central sensitization [9,10,11,12]. Over the last two decades, microglia have been found to be activated in various kinds of pain, including migraine. Activated microglia release a large number of inflammatory cytokines, such as interleukin (IL-1β) and tumor necrosis factor (TNF-α), that can bind to corresponding receptors on the membrane of neurons, thus regulating the neuron activity and promoting the transmission and amplification of pain signals [13,14]. Microglia, associated cytokines, and central microglia–neuron interactions enhance central sensitization and are important regulatory factors in chronic pain [11,15]. Moreover, multiple studies have found that inhibition of microglial activation or inflammatory cytokines can significantly relieve CA and central sensitization (i.e., depress the levels of c-Fos and calcitonin gene-related peptide (CGRP)) [15,16,17,18]. As such, microglial activation has become a new target in understanding the pathophysiological mechanisms behind migraines.

There has been a lot of clinical and experimental evidence that acupuncture has achieved efficacy in the prevention and treatment of migraine [19,20,21]. Fengchi (GB20), located in the depression of the upper end of sternocleidomastoid muscle and trapezius muscle, belongs to the point of the Foot–Shaoyang gallbladder meridian. Migraine pain is located in the head and temporal part, where the Foot–Shaoyang gallbladder meridian passes through. According to the acupoint selection principle of “meridian syndrome differentiation” in traditional Chinese medicine, Fengchi acupoint is an effective and high-frequency acupoint for clinical treatment of migraine [22]. Previous studies have found that EA at GB20 in migraine rat models can ameliorate migraine-like symptoms (i.e., reflective of CA) via inhibition of the trigeminal pain pathway, which is mediated by CGRPergic and serotoninergic neurons [23,24,25]. These data suggest that acupuncture in migraine can modulate neurons via the trigeminal pain pathway. Whether microglia activation and its inflammatory response are involved in anti-migraine effects of acupuncture is unknown. To explore the microglial mechanisms of EA in migraine, we hypothesized that EA at GB20 could ameliorate migraine central sensitization by suppressing microglial activation and the subsequent release of inflammatory cytokines and their receptors in neurons in a migraine rodent model.

To confirm this hypothesis, repeated dural electrical stimulation (DES)-treated rats were used and cephalic cutaneous mechanical sensitivities were assessed by electronic von Frey in conscious animals. To determine whether EA can relieve central sensitization by mediating microglial activation in the trigeminal nucleus caudalis (TNC), morphological changes of microglia cells were observed and quantified using immunofluorescence. The expressions of the microglial biological marker, Ibal-1, and proinflammatory cytokines (e.g., IL-1β and TNF-α) in the TNC were evaluated by Western blot and real-time polymerase chain reaction (rt-PCR). For neurons in the TNC, the expression of TNF-α receptor (R) and IL-1βR, and receptors for TNF-α and IL-1β were all assessed by immunofluorescence and Western blot. Finally, to investigate whether microglial inhibitors also have an acupuncture-like role, we examined the behavioral hypersensitivity and expression of c-Fos in neurons and Iba-1, IL-1β, and TNF-α protein in the TNC.

## 2. Materials and Methods

### 2.1. Animals

Individual male Sprague–Dawley rats (210 ± 10 g; Pengyue Experimental Animal Breeding Co., Ltd., Jinan, China) were acclimated to a 12 h light–dark cycle (lights on at 08:00 am); water and food were provided as well. After a one-week acclimation period, the experiment was begun. All experimental procedures are in accordance with the national Guidelines for the Care and Use of Laboratory Animals (State Council of China, 2013). Our study was reviewed and approved by the Ethics Committee of our institution. At the end of the experiment, all rats were sacrificed by intraperitoneal injection of 10% chloral hydrate (15 mL/kg), followed by cervical dislocation/aortic perfusion. All surgeries were performed under anesthesia (loss of consciousness, loss of sensation and response to painful stimuli anywhere in the body), which reduced pain.

### 2.2. Dura Mater Electrode Implantation

As described previously [23], rats were anesthetized by intraperitoneal injection of sodium pentobarbitone (60 mg/kg); Sigma-Aldrich, St. Louis, MO, USA) and restrained on a stereotaxic instrument. Two electrodes (JiAnDeEr Ltd., Beijing, China) were carefully implanted on the dura near the superior sagittal sinus. The fixed positions were 4 mm anterior to the posterior fontanelle and 6 mm posterior to the posterior fontanelle at the midline suture. Then, dental cement fixation and penicillin (40 thousand IU/100 g) anti-infection prophylaxis was used for 3 days following surgery. After 1 week of postoperative recovery, the experiment was continued.

### 2.3. Experimental Design

#### 2.3.1. Experiment 1

**Figure 1:**
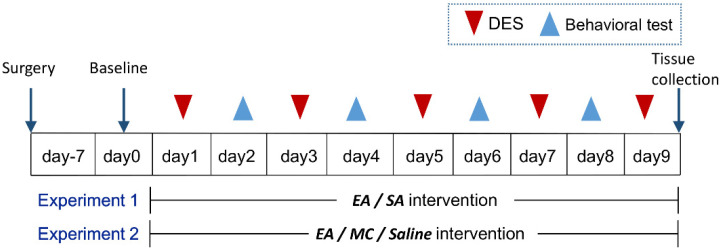
Schematic representation of the experimental protocol. After the post-surgery recovery (7 days), rats received repeated DES every 2 days (day 1, 3, 5, 7 and 9). EA at GB20/sham-acupoint (Experiment 1) and MC/saline systemic administration (Experiment 2) were performed every day from day 1 to 9. Facia mechanical threshold were assessed on day 0 (baseline), 2, 4, 6, and 8 by electronic von-Frey. Tissue and specimen collection were conducted on day 9 after the last intervention.

To determine the antimigraine effect of EA on the microglianeuron network in the TNC of rats, 80 animals were randomly, equally divided into 4 groups: model (only DES), EA (DES + EA at GB20), SA (DES + EA at sham-acupuncture point), and control (only electrode implantation). Except for the control groups, rats of the other three groups were administered repeated DES by a matching wire connected to a stimulator every other day for a total of five sessions (days 1, 3, 5, 7, and 9). After DES, rats of the EA and SA groups were given EA at bilateral GB20 and sham-acupoint, respectively, once per day for a total of nine sessions. CA was determined on days 0 (baseline), 2, 4, 6, and 8. After the experiment, day 9, all rats were sacrificed for follow-up assay. Figure 1 illustrated the schematic diagram of the experiment design.

#### 2.3.2. Experiment 2

To investigate whether the inhibition of microglial activation has an acupuncture-like role in the migraine central sensitization and verify the therapeutic mechanisms of EA, minocycline (MC; SigmaAldrich, St. Louis, MO, USA), a microglia inhibitor, was systemically administered to specifically inhibit microglial activation. All 40 rats were divided into 4 groups after post-surgery recovery: model (DES), EA (EA + DES), MC (MC + DES), and saline (saline + DES) groups. All rats received DES, as described above. Rats of the MC group were injected with 150 mg/kg of MC immediately after DES once per day from days 1–9 for a total of nine sessions, as described previously [15]. The Saline group received an equal volume of saline. All animals were sacrificed on day 9 for follow-up assay. The study timeline of the study is depicted in Figure 1.

### 2.4. Establishment of Recurrent Migraine Rat Model

A rat model of recurrent migraine was induced in conscious rats by repeated DES, as described previously [23,24]. Except for the control group, all rats were given repeated DES. Rats were stimulated using an electrical stimulator (YC-2, Chengdu Instrument, Chengdu, China) and treated every other day (days 1, 3, 5, 7 and 9). The parameters of electric stimulus were set as: waveform (square wave pulse), pulse duration (0.5 ms), current intensity (1.8~2.0 mA), stimulus frequency (20 Hz), and stimulus duration (10 min). The rats of the control group were only connected to the electrical stimulator, but without stimulus current.

### 2.5. EA Intervention

Rats of the EA and SA groups were restrained in custom-made rat immobilization devices, ten to fifteen minutes after DES, in order to adequately expose their heads and necks for treatment, which was done every day. Disposable stainless filiform needles with 0.25 mm diameter and 25 mm length were used in the experiment. Rats in the EA group were given bilateral GB20 acupuncture points whose position was 3 mm from the center point between the two ears at a depth of 1 cm to the opposite eye; the SA group was given bilateral sham-acupoint (located 10 mm above iliac crest) based on previous studies. An acupoint nerve stimulator (HNAS-200, Nanjing Jisheng Medical Technology Company, Nanjing, China) was used in experiment. Electrical stimulation parameters were amplitude-modulated waves with a frequency of 2/15 Hz. The current intensity was adjusted according to the rat’s response (local muscle contraction, rats in a quiet state without pain and struggle) and ranged from 0.5~1.0 mA. The output time of electrical stimulation was set to 15 min. During the whole course of treatment, the control and model groups were kept in the fixture, but no measurements or stimulations were administered. All rats were conscious during the intervention.

### 2.6. Facial Mechanical Threshold by Electronic von Frey

The facial withdrawal thresholds to mechanical stimuli were conducted under blinded conditions. All rats in the same feeding room were stimulated on days 0 (baseline), 2, 4, 6, and 8 during the 08:00–10:00 time period.

The rats were restrained in a plastic cylinder (25 cm long and 8 cm inner diameter) for 30 min, and an electronic von Frey anesthesia instrument (model 2390; IITC Life Science Inc., Woodland Hills, CA, USA) was used to test the facial withdrawal threshold of mechanical stimulation. The specific operation methods and precautions have been described in our previous work [24]. The rats were stimulated three times on each side of the face, alternately, with an interval of approximately 8 s.

### 2.7. Immunofluorescence and Image Analyses

Immunofluorescence was used to detect microglial markers (Ibal-1), IL-1β, TNF-α, and their receptors and c-Fos. Tissue sections were made following routine perfusion sampling, dehydration, and fixation with paraffin-embedding. Coronal sections of Iba-1-labeled microglia are 8 µm thick; other labels are 5 µm thick. A cryostat (CM3050S; Leica Inc., Wetzlar, Germany) was used to obtain the TNC and then treated with Cy3 goat anti-rabbit secondary antibodies for 30 min at 25 °C, followed by incubation with relevant antibodies for 1 h at 37 °C. Double labeling was performed using two primary antibodies obtained from different genera. After washing, the slices were incubated at 37 °C for 0.5 h with a solution containing species-specific secondary antibodies conjugated to Alexa 488 (Beyotime, Shanghai, China). After washes, all sections were counterstained with DAPI.

The slides were scanned using Pannoramic MIDI (3DHISTECH; Budapest, Hungary) and measured using CaseViewer 2.3 software (3DHISTECH; Budapest, Hungary) at 40× magnification. The cell number and fluorescence intensity were observed by a morphological image analysis instrument (JD801, JSJD Tech Inc., Nanjing, China) as well as Image J (https://imagej.net/, accessed on 6 December 2020) in squares. The image analysis was performed by a blinded observer.

### 2.8. Western Blot

The microglial markers (Ibal-1), IL-1β, TNF-α, and their receptors in the TNC were detected by Western blot. First, the total cellular protein was extracted from tissue specimens using a lysis buffer containing a 1× protease inhibitor cocktail. Then, the proteins were quantified using the BCA protein assay kit (P0012S; Beyotime, Shanghai, China). Subsequently, the same amounts of protein were resolved on a 10% SDS-PAGE and electroblotted onto polyvinylidene difluoride membranes. Then, membranes were sealed in 5% nonfat milk for 2 h, washed 3 times with PBS, and incubated with the primary antibodies for 12 h (the specific dilution ratio is shown in the Antibodies section). After 24 h, the samples were washed 3 times with PBS and then incubated for 1.5 h with appropriate secondary antibodies. The protein bands were detected with the ECL hypersensitive luminescence kit (no. 34094, ThermoFisher Scientific, Waltham, MA, USA), and the digital images were acquired using an automatic exposure instrument (JS-1070P; Shanghai Peiqing Technology Co., Ltd., Shanghai, China) and analyzed using Image J.

### 2.9. Real-Time PCR

Using TRIzol reagent (no. 15596026; Life Technologies, Frederick, MD, USA) the total RNA was extracted from exosomes and cells. cDNA was synthesized using the PrimeScript RT Reagent Kit with gDNA Eraser (no. RR047A; Takara Bio Inc., Shiga, Japan). Then, quantitative PCR (qPCR) was conducted using the Novostart SYBR qPCR SuperMix Plus (E096-01B; Novoprotein, Shanghai, China). PCR amplification was conducted using the Fluorescence Quantitative PCR Instrument (PikoReal 96; ThermoFisher Scientific) with β-actin serving as a reference gene. PCR cycle conditions were as follows: first 95 °C for 1 min, then 95 °C for 20 s, 60 °C for 1 min for 40 cycles. All experiments were conducted in triplicate. The amplification primers were:
β-actinForward primer 5, -CCCATCTATGAGGGTTACGC-3;
Reverse primer 5, -TTTAATGTCACGCACGATTTC-3;Iba-1Forward primer 5, -CTCCGAGGAGACGTTCAGTT-3;
Reverse primer 5, -TTGGCTTCTGGTGTTCTTTGT-3;TNF-αForward primer 5, -GGGCCACCACGCTCTTCTGT-3;
Reverse primer 5, -GGCTACGGGCTTGTCACTCG-3;IL-1βForward primer 5, -AGGCAGTGTCACTCATTGTGG-3;
Reverse primer 5, -TAGCAGGTCGTCATCATCCC-3;

The 2^−^^ΔΔCT^ relative quantitative method was used to calculate the relative abundance of each target gene mRNA.

### 2.10. Antibodies

The antibodies used for immunostaining were as follows:

1. Rabbit anti-Iba-1(Iba-1 as microglial marker, 1:400,019-19741, Wako Chemicals, VA, USA);

Mouse anti-c-Fos (1:200, ab190289, Abcam, Cambridge, UK);
Mouse anti-TNFRII (1:200, SC-8041, Santa Cruz, CA, USA);
Mouse anti-IL-1RI (1:400, SC-393998, Santa Cruz, CA, USA);
Rabbit anti-NeuN (neuronal marker, 1:400, ab236870, Abcam, Cambridge, UK).

The antibodies used for Western blot were as follows:

2. Rabbit anti-TNF-α (1:500, bs-2081R, Bioss, Beijing, China);

Mouse anti-TNF-αRII (1:500, Sc-8041, Santa Cruz, CA, USA);
Rabbit anti-IL-1β(1:500, bs-6319R, Bioss, Beijing, China);
Mouse anti-IL-1βRI (1:500, Sc-393998, Santa Cruz, CA, USA);
Mouse anti-Iba-1 (microglial calcium-binding protein, 1:400, Sc-32725, Santa Cruz, CA, USA).

### 2.11. Statistical Analysis

All experiment data are in the form of mean ± standard deviation. Data were analyzed using one-way analysis of variance. The homogeneity of variance of the data was analyzed by Bonferroni or the heterogeneity of variance of the data was analyzed by Tamhane, or the nonparametric test of two independent samples was analyzed by the Mann–Whitney test. Statistical evaluation was performed using SPSS software (SPSS version 20.0; IBM Corporation, Armonk, NY, USA). Statistical significance was considered when *p* < 0.05.

## 3. Results

### 3.1. EA Attenuated DES-Induced Facial Mechanical Threshold

**Figure 2:**
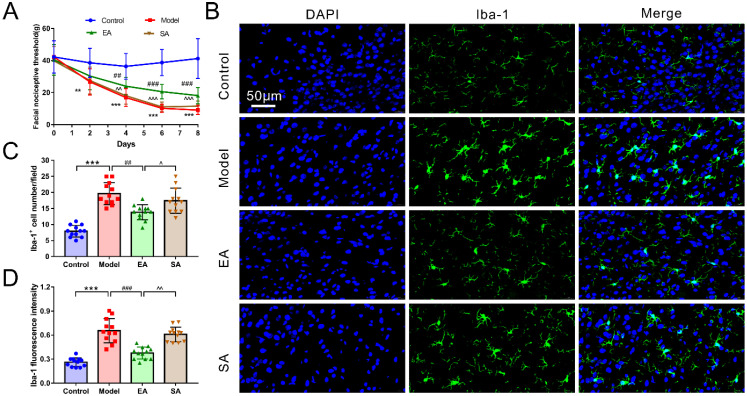
Facial nociceptive threshold and Iba-1-labeled microglia activation in the TNC among groups. (**A**) Facial nociceptive threshold among groups at all time points throughout the experiment (*n* = 20/time point/group); (**B**) Images of microglia labeled with Iba-1 immunofluorescence staining in TNC (400×, Scale bar = 50 μm, *n* = 4, three sections/rat); (**C**) Number of Iba-1-labeled microglia cell (*n* = 4, three sections/rat); (**D**) Iba-1-labeled microglia fluorescence intensity (*n* = 4, three sections/rat). One-way ANOVA followed by post hoc Tamhane test. Data are mean ± SD. ** *p* < 0.01, *** *p* < 0.001, model versus control. ## *p* < 0.01, ### *p* < 0.001, EA versus model. ^ *p* < 0.05, ^^ *p* < 0.01, ^^^ *p* < 0.001, SA versus EA.

Figure 2A plots the behavioral results of rats among groups. It can be seen that there was obviously no difference in facial nociceptive thresholds between groups before the DES baseline (*n* = 20, *p* = 0.851). DES was found to significantly reduce the facial nociceptive threshold across subsequent time points (model & control: days 2, *p* = 0.001; days 4, 6 and 8, *p* < 0.001). Interestingly, EA-GB20 markedly reversed these effects at all time points in the EA group, except for day 2 (model & EA: day 2, *p* = 0.686; day 4, *p* = 0.001; days 6 and 8, all *p* < 0.001). Nonetheless, the sham-acupoint of the EA group did not change sensory thresholds, as indicated by a non-significant difference threshold (model & SA: all *p* > 0.05 among four time points).

### 3.2. EA Attenuated DES-Induced Microglial Activation and Inflammatory Factor Release in the TNC

Microglia in the TNC were immunolabeled with Iba1. Representative microphotographs and results depicting Ibal-1 immunopositive cells in the TNC are shown in Figure 2. As shown in Figure 2B–D, after repeated DES, microglia displayed an activated phenotype, manifested by an apparent increase in the cell number and fluorescence intensity (reflecting the increased cell density and cell body) of the model group (model and control: Iba1 + cell number/fluorescence intensity, all *p* < 0.001).

**Figure 3:**
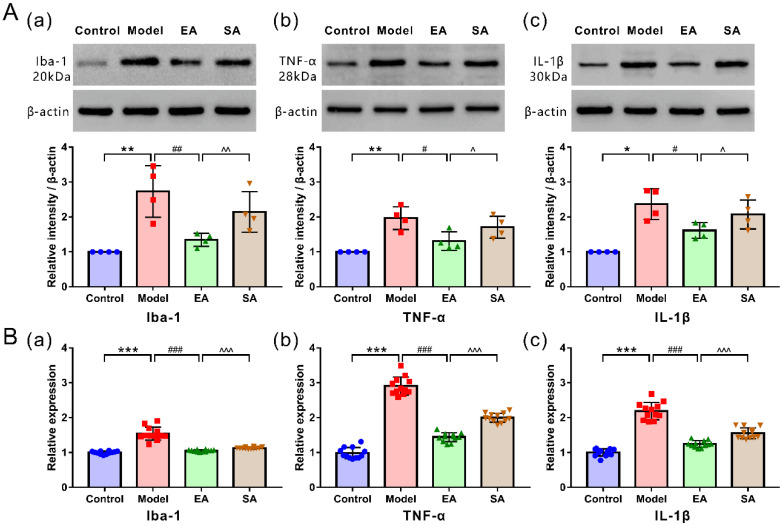
Levels of protein and mRNA expression of Iba-1, inflammatory cytokines (IL-1β and TNF-α) in the TNC among groups. (**A**) Western blot analysis and band density quantification of Iba-1 and inflammatory cytokines (IL-1β and TNF-α) (*n* = 4). (**B**) mRNA expression of Iba-1, inflammatory cytokines (IL-1β and TNF-α) by RT-qPCR (*n* = 12). Protein expression: Mann–Whitney nonparametric test; mRNA expression: One-way ANOVA followed by post hoc Tamhane test. Data are mean ± SD, * *p* < 0.05, ** *p* < 0.01, *** *p* < 0.001, model versus control; # *p* < 0.05, ## *p* < 0.01, ### *p* < 0.001, EA versus model; ^ *p* < 0.05, ^^ *p* < 0.01, ^^^ *p* < 0.001, SA versus EA.

The protein (Figure 3(Aa)) and mRNA (Figure 3(Ba)) expression levels of Ibal-1 and proinflammatory cytokines TNF-α (Figure 3(Ab,Bb)) and IL-1β (Figure 3(Ac,Bc)), as assessed using WB and PCR, were also higher in the model group (model & control: protein expression: Ibal-1/TNF-α, *p* = 0.002; IL-1β, *p* = 0.02; mRNA expression: Ibal-1/TNF-α/IL-1β, all *p* < 0.001). We assessed whether EA affected DES-induced microglial activation and inflammatory factor release in the TNC. As expected, microglia were significantly depressed (decrease in cells’ number and fluorescence intensity) (Figure 2; EA & model: Iba1^+^ cell number, *p* < 0.001; fluorescence intensity, *p* = 0.001), and the protein and mRNA expressions of Iba-1, TNF-α, and IL-1β were remarkably reduced in the EA group (Figure 3; EA & model: protein expression: Ibal-1, *p* = 0.004; TNF-α, *p* = 0.01; IL-1β, *p* = 0.025; mRNA expression: Ibal-1/TNF-α/IL-1β, all *p* < 0.001).

### 3.3. EA Attenuated DES-Induced High Expression of Inflammatory Factor Receptors in TNC Neurons

**Figure 4:**
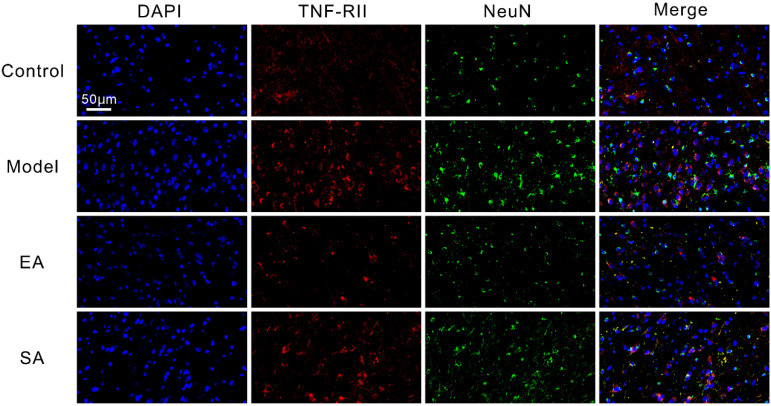
Representative immunofluorescent inflammatory factor receptor TNF-αRII in TNC. Shown are NeuN (green) and inflammatory factor receptor TNF-αRII (red) double-labeled cells in the TNC (400×, Scale bar = 50 μm, *n* = 4, three sections/rat).

**Figure 5:**
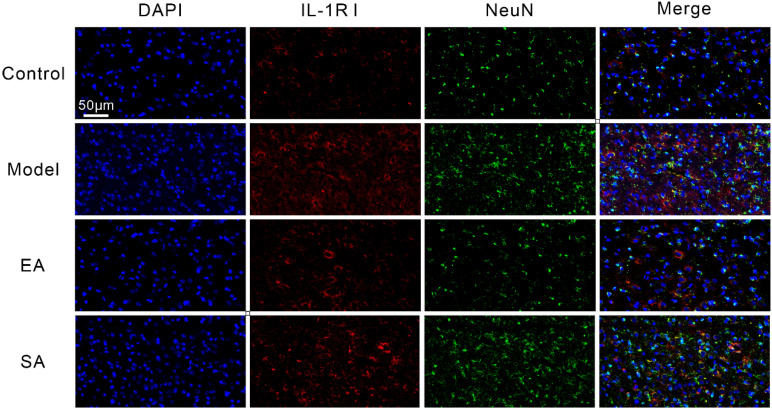
Representative immunofluorescent and protein expression of inflammatory factor receptor IL-1βRI in TNC. Shown are NeuN (green) and inflammatory factor receptor IL-1βRI (red) double-labeled cells in the TNC (400×, Scale bar = 50 μm, *n* = 4, three sections/rat).

**Figure 6:**
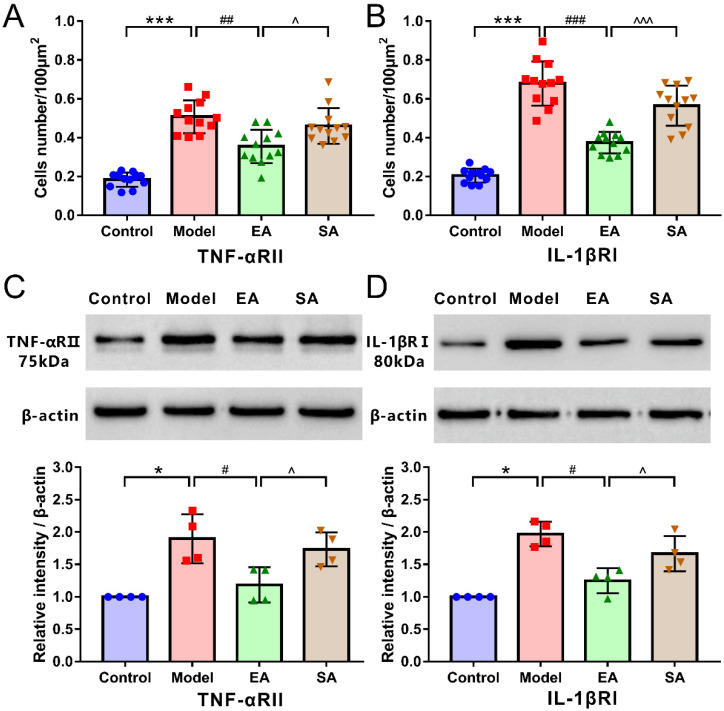
Quantification of inflammatory factor receptor positive neurons and protein expression level in the TNC. Quantification of the numbers of TNF-αRII (**A**) and IL-1βRI (**B**) positive neurons in the TNC (*n* = 4/100 μm^2^); Representative Western blot analysis and band density quantification of TNF-αRII (**C**) and IL-1βRI (**D**) in TNC neurons (*n* = 4); Mann–Whitney nonparametric test. Data are mean ± SD. * *p* < 0.05, *** *p* < 0.001, model versus control. # *p* < 0.05, ## *p* < 0.01, ### *p* < 0.001, EA versus model. ^ *p* < 0.05, ^^^ *p* < 0.001, SA versus EA.

As shown in Figure 4 and Figure 5, double-label immunofluorescence displayed inflammatory factor receptors TNF-αRII and IL-1βRI that were co-located with TNC neurons. The TNC sections of the model group contained more inflammatory factor receptor-positive neurons than sections from the control group (Figure 6A,B; model & control: TNF-αRII/IL-1βRI, both *p* < 0.001); however, these results can be reversed by repeated EA-GB20 by detecting a reduction in the number of receptor-positive neurons (Figure 6A,B; model & EA: TNF-αRII: *p* = 0.001; IL-1βRI: *p* < 0.001), although they were not attenuated in the SA group (model & SA: *p* > 0.05 for both).

Figure 6 shows the protein expression levels of inflammatory factor receptors TNF-αRII (Figure 6C) and IL-1βRI (Figure 6D) (assessed using WB) were higher in the model group (model & control: TNF-αRII/IL-1βRI, both *p* = 0.014;). EA-GB20 significantly reduced the protein expression of inflammatory factor receptors in the TNC (model and EA: TNF-αRII/IL-1βRI, both *p* = 0.021), and there was no significant variation in EA at the sham acupoint (model & SA: TNF-αRII/IL-1βRI, both *p* > 0.05).

### 3.4. Minocycline Has a Partial Acupuncture-Like Effect in Migraine Central Sensitization: Relieving DES-Induced Facial Mechanical Threshold and TNC Activation

**Figure 7:**
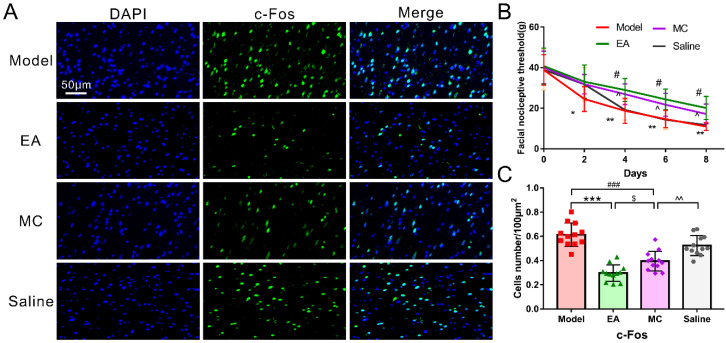
The acupuncture-like role of minocycline in the migraine central sensitization. (**A**) Images of c-Fos-immunoreactive neurons in TNC in each experimental group (400×, Scale bar = 50 μm). (**B**) Facial nociceptive threshold among groups (*n* = 10). (**C**) The density of c-Fos positive neurons in TNC in each experimental group (*n* = 4, three sections/rat). One-way ANOVA followed by post hoc Tamhane test. Data are mean ± SD, * *p* < 0.05, ** *p* < 0.01, *** *p* < 0.001, model versus EA. $ *p* < 0.05, EA versus MC. # *p* < 0.05, ### *p* < 0.001, MC versus model. ^ *p* < 0.05, ^^ *p* < 0.01, saline versus MC.

We aimed to verify whether inhibition of microglia activation could have an acupuncture-like effect in our migraine model and verify the therapeutic mechanisms of acupuncture. As expected, MC administration also depressed the activation of the trigeminal neurovascular system, which was reflected by the decrease in c-Fos positive neurons in the TNC (Figure 7A,C; MC & model: *p* < 0.001). Figure 7B shows MC administration significantly reversed the DES-induced facial mechanical threshold on days 4, 6, and 8 (MC & model: days 4, *p* = 0.026; days 6, *p* = 0.034; days 8, *p* = 0.019), but not on day 2 (MC & model: *p* > 0.05). Compared with the EA group, the facial mechanical threshold in the MC group decreased at each time point, but there was no significant difference between the two groups (MC & EA: days 2, 4, 6, and 8, all *p* > 0.05).

**Figure 8:**
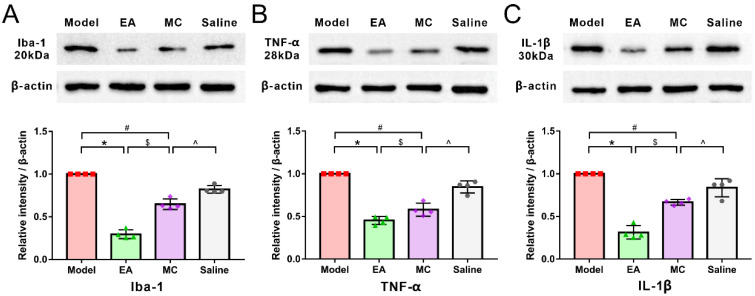
The acupuncture-like effect of minocycline on microglia activation in migraine rat. Western blot analysis and band density quantification of Iba-1 (**A)**, IL-1β (**B**) and TNF-α (**C**) (*n* = 4). Mann–Whitney nonparametric test. Data are mean ± SD. * *p* < 0.05, model versus EA. $ *p* < 0.05, EA versus MC. # *p* < 0.05, MC versus model. ^ *p* < 0.05, saline versus MC.

In immunofluorescence (Figure 7A,C)and WB experiments (Figure 8), MC inhibited the activation of c-fos neurons induced by repeated-DES and downregulated the protein expressions of Iba-1, TNF-α, and IL-1β (MC & model: c-Fos, *p* < 0.001; Iba-1, *p* = 0.029; TNF-α, *p* = 0.029; IL-1β, *p* = 0.021. MC & EA: c-Fos, *p* = 0.033; Iba-1, *p* = 0.031; TNF-α, *p* = 0.029; IL-1β, *p* = 0.029), mimicking acupuncture-like effects; but there is a significant difference compared with the EA group, indicating that the mechanism of acupuncture antimigraine effect is partly mediated by inhibiting the activation of microglia.

## 4. Discussion

Based on the traditional “neuron drive theory”, we have previously confirmed that EA-GB20 could alleviate migraine central sensitization by altering the trigeminal vascular system and descending pain modulation system [23,24,25]; however, a new “microglial involvement theory” is gaining attention within the scientific community [12,13]. The present study aimed to determine if EA-GB20 could improve central sensitization by inhibiting microglial activation of the TNC in a migraine rodent model and explore the potential mechanism of EA. We found that improvements in central sensitization and CA were at least partially because EA-GB20 inhibited microglial activation.

### 4.1. EA at GB20 Alleviated Migraine Central Sensitization by Inhibiting Microglial Activation

CA, caused by harmless cutaneous stimulation, has been considered as an independent predictor of migraine chronicity and clinical marker of central sensitization [2,26]. Facial/cephalic CA indicates that the two-order trigeminovascular neurons are sensitive, which is a sign of trigeminal neurovascular system activation [27]. The mechanism of central sensitization has not been elucidated, and previous studies have focused on the neurons in the trigeminal neurovascular and descending facilitation/inhibition systems [7,28]. Over the last 20 years, microglial activation-mediated neuropathic pain, including migraine, has garnered attention, and “microgliapathic pain” has become a novel research area of interest [13,15].

Microglia are the main immune cells of the central nervous system and have the properties of macrophages and are widely distributed in the brain and spine/medulla oblongata. The traditional theory is that microglia are only involved in myelin formation and the maintenance of nerve homeostasis, and they have a role in nutrition and support; they do not, however, participate in the transmission of pain information and algesthesia modulation [29]. Previous research suggests that microglial activation affects the evolution of neuropathic pain, including migraine [11,13,30,31]. During migraine attack, trigeminal nerve ending fibers are stimulated to release neuropeptides such as calcitonin gene-related peptide (CGRP) and substance P (SP), which cause neurogenic inflammation of the meninges. Nociceptive information is transmitted via the trigeminal ganglia to secondary neurons (TNC) and higher structures, inducing increased neuronal sensitivity and excitability and at the same time activating microglia [27,32]. First, activated microglia undergo morphological and quantitative changes, showing enlarged cell bodies and density with decreasing protrusion length and complexity. Subsequently, hyperactive microglia release a large number of inflammatory mediators and cytokines, for example, IL-1β and TNF-α. These inflammatory factors can bind to the corresponding receptors on the neuron cell membrane, causing various plastic modifications of neuronal synapses and networks at the molecular and structural levels, promoting the transmission and amplification of pain signals, which further exacerbate central sensitization [10,13,16].

Accordingly, microglia activation has become an important regulatory factor in the progression of chronic migraine.

Clinical observations have found that cerebrospinal fluid TNF-α levels are elevated in chronic migraine [33] and that microglial inhibitors can inhibit migraine attack [34]. Recent experimental studies have demonstrated that microglia are significantly activated in migraine models, which are manifested as changes in cell morphology, increased number, and enlarged cell body, etc. In addition, the amount of microglial activation markers (Iba-1), IL-1β, and TNF-α were apparently increased in migraine rodent models, as well as reduced facial mechanical pain threshold. Moreover, blocking of microglia activation and/or inflammatory factors’ release can reduce mechanical hyperalgesia [18,35], blood–brain barrier permeability [15], and CGRP and c-Fos expression [16].

Consistent with previous research, our study found that after repeated DES, the facial mechanical threshold mediated by central sensitization decreased significantly. Microglia in the TNC were activated (demonstrated by morphological and quantitative changes) and accompanied by upregulated expression of Ibal-1, IL-1β, and TNF-α; however, EA at GB20 can significantly reverse these effects, suggesting that EA at GB20 reduced the facial withdrawal threshold by inhibiting microglial activation and preventing the release of inflammatory cytokines. We further question how microglial activation and release of inflammatory factors participate in neuron activation and pain regulation.

### 4.2. GB20 Can Regulate the Expression of Inflammatory Factors Receptors on Neurons

Research has demonstrated that TNC neurons have inflammatory cytokine receptors that can specifically bind to the inflammatory cytokines secreted by microglia after activation, serving as a link between microglia and neurons [14]. This microglia–neuron interaction can promote neuronal activity, promote the transmission and amplification of pain information, and ultimately induce central sensitization and the formation of chronic pain [11]. Recent studies have found that the latest member of the tachykinin family encoded by the Tac4 gene in the tripeptide system, heme-1 (HK-1), may be involved in microglia–neuron interactions under physiological and inflammatory conditions, and mediates pain in the trigeminal nervous system [36].

The upregulation and phosphorylation of the neuronal glutamate receptor subunit NMDAR is also involved in microglial inflammatory responses and is a major regulator of microglia–neuron interactions [14]. Therefore, although the understanding of their roles is currently incomplete, they appear to be functionally interconnected and form one or more identifiable signaling pathways, and biological signaling pathways related to microglia–neuron interactions remain to be seen in further research.

Immunofluorescence double labeling has previously demonstrated that inflammatory factor receptors TNF-αR and IL-1βR were specifically expressed in neurons, not glial cells, and that the maintenance of mechanical hyperalgesia was positively correlated with their increased expression in neurons [11,13]. Nevertheless, cytokine inhibitors or receptor antagonists significantly reversed the behavioral hypersensitivity and c-Fos and CGRP levels in the TNC [13]. We found that TNF-αR and IL-1βR were co-located with TNC neurons. An upregulation of factor receptor expression was also observed after DES in the TNC, but the enhanced expressions of TNFαR and IL-1βR induced by DES were apparently decreased in the EA group. These results suggest that the mechanism of acupuncture in antimigraine central sensitization may be through the regulation of the expression of inflammatory factor receptors on neurons.

### 4.3. Minocycline, a Microglia Inhibitor, Can Exert Part of Acupuncture-like Effects

MC is widely recognized as a microglia inhibitor with potential efficacy in the treatment of migraine. The administration of MC can inhibit the activation of microglia and the subsequent release of inflammatory factors and relieve allodynia [15,29]. To confirm whether the inhibition of microglial activation could have an acupuncture-like effect in our migraine model and further verify the therapeutic mechanism of acupuncture, MC was systemically administered to inhibit microglial activation. We found that systematic administration of MC after DES increased the mechanical threshold and alleviated pain after day 4; it also decreased the upregulation of c-Fos, Iba-1, and the expression of inflammatory factors (TNF-α and IL-1β) in the TNC. Mechanical threshold indexes demonstrated no significant difference at each time point compared with the EA group, but the activation of c-Fos neurons and the protein expression of Iba-1 and inflammatory factors (TNF-α and IL-1β) in the TNC were significantly different, suggesting that the antimigraine mechanism of acupuncture may be partly mediated by inhibiting microglial activation. In this study, we provided the first preliminary evidence that the EA can mediate antimigraine central sensitization through microglia activation, inflammatory response, and the expression of cytokine receptors in TNC neurons. The signal transduction pathway after the binding of inflammatory factors and factor receptors and the exact molecular mechanism of microglia–neuron interaction needs further studied.

## 5. Conclusions

Our preclinical results demonstrate that EA-GB20 treatment can significantly decrease DES-induced behavioral hypersensitivity by inhibiting microglial activation and subsequent inflammatory factors’ release and the expression of cytokine receptors in TNC neurons in migraines. Furthermore, inhibition of microglia by MC may have an acupuncture-like role in migraines, as manifested by ameliorated mechanical hyperalgesia and decreased c-Fos expression in the TNC. EA-GB20 treatment improves central sensitization in CA and migraine, possibly by inhibiting microglia activation and the subsequent release of inflammatory cytokines and their receptors in TNC neurons. In summary, our research provides new evidence for the microglial reactivity to participate in the central sensitization of migraine and confirms that the effective mechanism of EA against migraine central sensitization is partly mediated by inhibiting microglial activation and its inflammatory reaction. This ongoing research aims to explore the intracellular and extracellular signal transduction involved in the microglia–neuron interaction and investigate the underlying mechanisms behind the efficacy of EA in the treatment of migraine symptoms.

## Data Availability

Not applicable.
